# The interaction between rs 3,807,992 genotypes with the dietary inflammatory index on Leptin, Leptin resistance, and Galectin 3 in obese and overweight women

**DOI:** 10.1186/s12902-022-01136-x

**Published:** 2022-09-23

**Authors:** Farideh Shiraseb, Mena Farazi, Niloufar Rasaei, Cain C. T. Clark, Shahin Jamili, Khadijeh Mirzaei

**Affiliations:** 1 Department of Community Nutrition, School of Nutritional Sciences and Dietetics, Tehran University of Medical Sciences (TUMS), P.O.Box: 14155-6117, Tehran, Iran; 2 Centre for Intelligent Healthcare, Coventry University, Coventry, CV1 5FB UK; 3 General Surgeon (Fellowship of Minimally Invasive Surgery), Department of Surgery, Shahid Beheshti University of Medical Sciences, Tehran, Iran; 4 Food Microbiology Research Center, Tehran University of Medical Sciences, Tehran, Iran

**Keywords:** Leptin, Leptin resistance, Galectin 3, Caveolin-1, Dietary inflammatory index, Interaction

## Abstract

**Objective:**

Obesity is related to increasing leptin and some inflammatory factors that are associated with low-grade inflammation. Moreover, several studies have shown Caveolin-1 (CAV1) genetic variations may be associated with dietary intake. The current study aimed to evaluate the interaction of CAV1 rs3807992 with types of the energy-adjusted dietary inflammatory index (EDII) in leptin, leptin resistance, and Galectin 3, as inflammatory factors.

**Methods:**

This cross-sectional study was carried out on 363 overweight and obese females. Dietary intake and DII were obtained from a 147-item food frequency questionnaire (FFQ). The CAV-1 genotype was measured using the PCR-RFLP method. Anthropometric values and serum levels of leptin and Galectin 3 were measured by standard methods.

**Results:**

Increased adherence to EDII in the interaction with CAV1 genotypes led to an increase in leptin level 79.15 (mg/l) (β = 79.15, CI = − 1.23,163.94, *P* = 0.04) in model 3, after controlling for further potential confounders. By contrast, adherence to EDII in the interaction with the genotype including risk alleles showed no significant interaction, even after adjustment in model 3 (β = 0.55, CI = − 0.99, 2.09, *P* = 0.48). Although, a marginal positive significant interaction was found between EDII and CAV1 genotypes on Galectin 3, after adjustment in model 3 (β = 31.35, CI = 0.13, 77.13, *P* = 0.05).

**Conclusions:**

The present study indicates that a high adherence of EDII and CAV1 genotypes containing risk alleles may be a prognostic factor and increase both leptin and Galectin3. However, it seems that the presence of interaction was not on leptin resistance. Further functional studies are necessary to elucidate the exact mechanism.

## Introduction

The obesity epidemic affects a large number of adults across the world [1], and it is causing a growing economic and health strain, with an extraordinarily high incidence rate among women of childbearing age. Indeed, a significant difference between the prevalence of obesity between females (29.8) and males (15.3%) has been reported [2]. Overweight and obesity are caused by the combination of various genetic, occupational, physiological, and eating habits, which result in inflammation [3]. Inflammation is affected by a variety of influences, including lifestyle, nutrition, and physical exercise, as well as genetic [4], whilst adiposity is linked to a higher incidence of non-communicable diseases (NCDs) [5, 6]. This systemic and adipose tissue inflammation, which causes increases in the production of leptin and pro-inflammatory cytokines, is one of the pathways that could illustrate the connection between obesity and the progression of NCDs, with the cause of chronic low-grade inflammation [7–9]. The overabundance of leptin released by adipocytes to the amount of body fat [10] has a key role in homeostatic regulations of feeding and energy balance thus body weight management and also insulin sensitivity [11–13]. One of the related factors to leptin and obesity is Galectin-3, which belongs to a family of animal lectins that bind beta-galactosides, and is distinguished from others by the inclusion of tandem repeats in its N-terminal region. Galectin 3, like the other members, lacks a conventional signal chain, but it is secreted by a nonclassical secretory pathway and can act in an autocrine or paracrine manner extracellularly that released in a constitutive or inducible manner by nearly all immune and inflammatory cell types. The role of Galectin 3 in the modulation of these cell functions has been shown in a wide body of work, especially inflammation, such that work in Galectin-3 deficient mice has shown that this protein plays a significant role in the inflammatory response [14, 15].

Another factor that could affect inflammation is genotype, where there seems to be a relationship between leptin and rs 3807992 genotypes (Caveolin 1 (CAV1)), such that leptin upregulates CAV1 expression [16]. CAV1 is a transmembrane scaffolding protein that controls essential cell functions such as proliferation, apoptosis, cell division, and transcytosis via a variety of signaling pathways and the progression of atherosclerosis and obesity. On the other hand, according to recent research, CAV1 expression is increased in human obesity, suggesting that leptin can play a crucial role [17]. CAV1 works in the same way as the suppressor of the cytokine signaling family of proteins, which are components of the classic negative feedback circuit [6]. They are upregulated by cytokines, and, as a result, they block cytokine-induced signaling pathways in the cell. Also, some studies have shown that genetic variations in CAV1 can interfere with other risk factors, such as dietary intake [18, 19]**.**

Diet, especially dietary patterns, plays a significant role in influencing obesity and circulating inflammatory markers in adults [8]. The Dietary Inflammatory Index (DII), developed by Shivappa et al., calculates the consumption of nutrient and non-nutrient components of the food and was recently introduced to measure the inflammatory properties of the diet, thus, it is considered as an overall picture of the inflammatory properties of the diet [20–22].To our knowledge, there is only one report, from a cross-sectional study conducted in females, which found an interaction between the DII score and CAV1 on leptin and Galectin 3. The DII must be evaluated in different demographic environments, because dietary habits differ throughout societies and can have an effect on the DII quality. Furthermore, since other influences, such as climate, lifestyle, and genetic history, vary around population settings, the association between the DII score and inflammation status, and interaction with genotype can be influenced. Therefore, this study aimed to evaluate the variants in *CAV1 (*rs 3,807,992 genotypes*) that could* interact with the DII index for serum Leptin, Leptin resistance, and Galectin-3 Levels in an obese and overweight Iranian population.

## Method

### Research design and study population

To perform a multicenter unregulated cross-sectional study, this observational study used a multistage cluster random sampling approach. The sample size was calculated according to the following formula: N = (([(Z1 − α + Z1 − β) × √1 − r 2]/r) 2 + 2), whit considering r = 0.35, β = 0.95, and α = 0.05. The participants in this sample were 363 healthy obese and overweight women between the ages of 18 and 48 who had a body mass index (BMI) of 25-40 at Tehran University of Medical Sciences, who were referred to urban health centers. Those with a medical history, opioid, or nicotine usage or alcohol consumption, thyroid disorder, diabetes mellitus, cardiovascular diseases (CVDs), malignancies, hepatic or renal conditions, menopause, lactation, breastfeeding, acute or chronic infections, people following weight loss diets and specific nutritional therapies, such as insulin, and cardiovascular diets, as well as weight fluctuations in recent months, and the consumption of dietary supplements in the preceding three months, or estimated energy intakes of more than 4200 kcal/d or less than 800 kcal/d, were excluded [23]. This study was approved by the Ethics Committee of Tehran University of Medical Sciences, Tehran, Iran (ID number: IR.TUMS.VCR.REC 1398.142). Before the start of the clinical screening tests, all patients were required to have written and informed consent. Grant ID: 97-03-161-41017).

### Anthropometric measurements

The InBody 770 scanner, a multi-frequency bioelectrical impedance analyzer, was used to determine body composition, comprising weight, BMI, fat mass, and fat-free mass (FFM) (Inbody Co., Seoul, Korea). This electrical impedance analyzer measures the resistance of body tissue to the passage of an electrical signal emitted by both hands and feet. If the current passes more quickly through certain parts of the body, the amount and ratio of body fat-free mass and fat mass can be calculated, as per the manufacturer’s instructions. Height was measured on a Seca scale stadiometer with an accuracy of 0.5 cm following standard appraoches. Waist circumference (WC) was measured in the narrowest part of the waist using a non-elastic tape with an accuracy of 0.5 cm while people were at the end of a normal exhalation. The largest part of the hip circumference (HC) was measured with an accuracy of 0.5 cm. The waist-to-hip ratio (WHR) was calculated as waist circumference (cm) divided by hip circumference (cm). The waist-to-height ratio (WHtR) was measured by dividing the waist circumference (cm) by the height (cm). A trained dietician places a measuring tape across the neck, beginning 1 inch from the point where the neck and shoulders meet, which may be the lower portion of Adam’s apple, to determine neck diameter (NC). Leptin resistance is measured by the following formula: leptin (mg/l)/BMI (kg/m^2^); this index assesses leptin levels when accounting for the influence of BMI [24].

### Dietary measurements and EDII calculation

To measure dietary consumption in the 12 months before the report, a 147-item semi-quantitative food FFQ was administered on a regular, weekly, annual, or yearly basis, a qualified researcher interviewed participants and collected their food intake number and frequency. A 147-item semi-quantitative FFQ validity and reliability has been confirmed by previous studies [25, 26]. Face-to-face interviews were used to assess dietary consumption using a standardized, reliable, and validated food-frequency questionnaire(FFQ) for Iran by a trained dietitian [27]. This evaluation was conducted by asking participants about the occurrence of food items consumed from a prepared list of foods. The servings and portion sizes reported by study subjects were converted to grams per day. Using household proportions, the portion sizes of the eaten items are translated to grams [28]. The Nutritionist IV program was then used to do a diet study (version 7; N-Squared Computing, Salem, OR, USA). EDII scores were calculated for all participants using FFQ-derived dietary data. The dietary data after energy adjustment were connected to a globally representative database that included food intake from eleven populations around the world, yielding a reliable mean and standard deviation estimation for each parameter [21].

The “standard global mean” was subtracted from the real dietary intake level, and the result was separated by the standard deviation to get z-scores. These z-scores were then translated into percentiles, with each percentile score being doubled and then subtracted by one to reduce the impact of ‘right skewing. ‘To achieve a food parameter-specific DII score for an individual, the based percentile score for each food parameter for each individual was compounded by the corresponding food parameter impact score [21]. After that, the average DII score was calculated by adding all of the food parameter-specific DII scores together. The higher the DII score, the more pro-inflammatory the diet, the lower the score, the more anti-inflammatory the diet [21]. The DII was determined using 29 food parameters from the FFQ (energy, starch, protein, total fat, monounsaturated fat, polyunsaturated fat, saturated fat, omega-3, omega-6 fatty acids, cholesterol, fiber, thiamin, riboflavin, niacin, vitamin B6, folic acid, vitamin B12, vitamin A, C, D, E and tea, onion, caffeine, iron, magnesium, selenium, zinc, and beta carotene).

### Biochemistry analysis

Participants were referred to the Nutrition and Biochemistry Laboratory of the School of Nutritional and Dietetics at Tehran University of Medical Sciences in this project. After a 10-12 hour overnight fast, venous blood samples were taken. Centrifuged for 15 minutes at 3000 rpm to isolate the EDTA anticoagulant plasma and serum samples, and the remaining blood was washed three times with 0.9 g/l NaCl solution. After serum isolation, the samples were instantly frozen at − 80 °C for laboratory testing. Pars Azmoon laboratory kits were used to measure triglyceride (TG), total cholesterol (TC), high-density lipoprotein (HDL), low-density lipoprotein (LDL), fasting blood pressure (FBS), and insulin levels in the blood (Pars Inc., Tehran, Iran). The active form of Galectin 3 was also measured using the ELISA-Quantikine kit’s enzyme-linked immunosorbent assay (ELISA) (R&D Systems, Minneapolis, MN). Industrial enzyme-linked immunosorbent assay kits were used to assess serum leptin concentrations (mg/l) (Mediagnost, Reutlingen, Germany). The homeostatic model assessment insulin resistance (HOMA-IR) was used to measure insulin resistance (mIU/ml), with the following equation: [fasting plasma glucose (mmol/l) and insulin (IU/l)] /22.5) [29]. After 15 minutes of rest, systolic blood pressure (SBP) and diastolic blood pressure (DBP) were measured three times with a mercury sphygmomanometer.

### Genotyping

Genotyping is the process of determining an individual's DNA, which was isolated from whole blood using a Mini Columns package to genotype the CAV1 polymorphisms (Type G; Genall; Exgene). CAV1 polymorphisms (rs3807992) in gene fragments were investigated using the polymerase chain reaction-restriction fragment length polymorphism (PCR-RFLP) technique (major allele G and minor allele A). The following primers were used for PCR: F:3′AGTATTGACCTGATTTGCCATG5′ R:5′GTCTTCTGGAAAAAGCACATGA-3′ In a DNA thermocycler, PCR reactions were carried out in a volume of 20 μl, comprising 1 l isolated DNA, 1 μl Forward primers, 1 μl Reverse primers, 7 μl purified water, and 10 μl Taq DNA Polymerase Master Mix. The DNA templates were denatured for 3 minutes at 94 degrees Celsius, followed by 40 cycles of denaturation at 94 degrees Celsius, annealing at 42-50 degrees Celsius, and elongation at 72 degrees Celsius for 2 minutes. Amplified DNA was digested overnight at 37 °C with Hin1II (NIaIII) restriction enzyme, then isolated on an agarose gel by electrophoresis (2%). Uncut homozygous AA (213bp), cut heterozygous GA (3 bands: 118, 95, and 213 bp), and cut homozygous GG genotypes of the CAV1 rs3807992 variant were identified (2 bands: 118 & 95 bp).

### Other covariates assessment

Participants' physical activity was assessed using the International Physical Activity Questionnaire (IPAQ), where its validity and reliability has been verified. This questionnaire contains seven questions, each of which has two sections (number of exercises per week and length) that indicate the participants' level of physical activity [30]. Other demographic characteristics, such as age, educational level, marital status, and income, were collected using standard questionnaires.

### Statistical analysis

The Kolmogorov-Smirnov method was conducted to test the data’s normality (*p* > 0.05). The Hardy-Weinberg Equilibrium deviation among CAV1, G32124A allele frequencies were determined using Pearson’s chi-square test. The discrepancies between the two groups of the median of EDII and genotype according to risk allele were assessed using an independent sample t-test, Chi-square test expressed as mean and standard error (SE), and for categorical variables as numbers and percentages, respectively. Analysis of covariance (ANCOVA) was utilized to account for confounders. Linear regression was used to assess the association between leptin, leptin resistance, and Galectin 3 with EDII and genotypes, presented as Β and 95% confidence interval (CI). A generalized linear model (GLM) was applied to obtain an estimate of interaction between EDII and Genotype on leptin, leptin resistance, and Galectin 3. SPSS v.25 program (SPSS Inc., IL, USA) was used for statistical analysis, and the significance level was set at a *P*-value < 0.05, whilst *P*-values 0.05, 0.06, and 0.07 were considered as marginally significant.

## Results

### Study population characteristics

General characteristics of participants, such as body composition, biochemical assessment, and others among lower vs higher than the median of EDII and genotypes, are presented in Table 1. A total of 363 women with BMI mean and SD 30.9 (3.90) kg/m^2^ were divided into two groups, based on EDII median (0.07) lower (*n*=172) and upper than (*n*=191) median. The range of EDII was -3.83 to 3.19, and 70.8% of the study population were married. The level of leptin in individual's serum had 27.7 (11.8) mg/l, and 4.02 (7.26) mg/l of Galectin3 (Table 1).

**Table 1:** Characteristics of the study population across rs 3,807,992 genotypes and median of EDII score in obese and overweight women (*n* = 363)

Variables	EDII median		***P***-value	***p***-value*	rs 3,807,992 genotypes		***p***-value	***p***-value*
	<0.07	> = 0.07			GG	AA + AG		
	***N*** = 172	***N*** = 191						
					***N*** = 75	***N*** = 198		
	Mean ± SE				Mean ± SE			
Age (years)	36.74 ± 0.79	35.67 ± 0.78	0.33	0.08	36.33 ± 0.89	35.58 ± 1.08	0.40	0.59
PA (MET-minutes/week)	1544.81 ± 208.08	205.33 ± 205.33	**0.007**	**0.04**	1062.62 ± 193.41	947.60 ± 234.38	0.64	0.70
Age of starting obesity	20.97 ± 0.88	23.31 ± 0.81	**0.10**	**0.05**	22.46 ± 0.84	22.38 ± 0.89	0.08	0.94
**Anthropometric variables**								
Weight (kg)	79.76 + 0.90	78.49 + 0.95	**0.05**	0.35	78.17 ± 0.70	76.51 ± 0.81	0.71	0.23
Height (cm)	161.84 + 0.84	160.60 + 0.93	0.68	0.35	161.41 ± 6.27	160.12 ± 5.97	0.24	0.56
BMI (kg/m^2^)	30.60 + 0.57	30.00 + 0.63	0.43	0.49	29.61 ± 0.39	30.51 ± 0.48	0.11	0.12
**Body composition**								
WC (cm)	94.45 + 1.18	93.23 + 1.25	0.26	0.49	96.66 ± 0.64	153.90 ± 3.47	0.13	0.52
HC (cm)	112.08 + 0.66	113.51 + 0.70	0.60	0.15	104.56 ± 0.20	103.97 ± 0.24	0.35	**0.06**
NC (cm)	36.26 ± 0.36	36.93 ± 0.39	0.54	0.22	36.46 ± 0.27	36.64 ± 0.32	0.30	0.44
WHR	0.92 + 0.00	0.93 + 0.00	0.11	0.24	0.921 ± 0.00	0.926 ± 0.00	0.12	0.23
WHtR	0.58 + 0.00	0.58 + 0.00	0.14	0.78	0.59 ± 0.00	0.60 ± 0.00	0.04	0.16
BFM (kg)	31.91 ± 0.38	33.06 ± 0.40	0.27	**0.05**	31.70 ± 0.31	31.84 ± 0.36	0.10	0.64
BFM (%)	41.14 ± 4.99	42.22 ± 5.71	0.91	**0.01**	40.31 ± 0.40	40.79 ± 0.46	0.10	0.71
FFM (kg)	47.87 ± 0.85	45.46 ± 0.90	**0.02**	**0.05**	46.32 ± 0.56	45.34 ± 0.65	0.68	0.52
SMM (kg)	26.30 ± 0.50	24.91 ± 0.53	**0.02**	**0.06**	25.41 ± 0.33	24.78 ± 0.38	0.64	0.46
SLM (kg)	45.10 ± 0.79	42.85 ± 0.84	**0.02**	**0.06**	43.64 ± 0.52	42.76 ± 0.61	0.73	0.55
BMC (kg)	2.68 ± 0.36	2.61 ± 0.32	0.09	**0.04**	2.67 ± 0.03	2.59 ± 0.04	0.33	0.35
Trunk fat(kg)	15.77 ± 0.18	17.29 ± 0.19	0.26	**0.01**	15.16 ± 0.14	15.62 ± 0.16	0.08	0.47
Visceral fat (kg)	14.62 ± 0.29	15.54 ± 0.31	0.69	**0.03**	14.58 ± 0.20	14.91 ± 0.23	**0.05**	0.25
FFMI	18.21 ± 0.16	17.58 ± 0.17	**0.004**	**0.01**	17.76 ± 0.13	17.60 ± 0.11	0.78	0.52
FMI	12.21 ± 0.16	12.85 ± 0.17	0.35	**0.01**	12.23 ± 0.11	12.38 ± 0.13	**0.07**	0.55
**Biochemical variables**								
FBS (mg/dL)	84.88 + 1.55	86.85 + 1.64	0.33	0.40	86.94 ± 1.01	86.85 ± 1.17	0.97	0.86
TC (mg/dL)	179.83 ± 5.49	174.49 + 5.83	0.75	0.51	180.44 ± 3.35	172.32 ± 3.88	0.18	0.17
TG (mg/dL)	123.11 + 12.10	132.78 + 12.85	0.65	**0.06**	110.96 ± 6.7	130.53 ± 7.85	0.08	**0.04**
HDL (mg/dL)	47.66 + 1.58	43.47 + 1.67	**0.04**	**0.07**	48.08 ± 1.04	45.20 ± 1.20	**0.02**	**0.04**
LDL (mg/dL)	99.60 + 3.92	92.87 + 4.16	0.44	0.25	95.83 ± 2.37	93.55 ± 2.75	0.23	0.20
**Categorical variables**								
Economic status								
Low level	51(62.2)	31(37.8)	**0.01**	**0.01**	28(32.6)	58(67.4)	0.31	0.20
Moderate level	72(43.4)	94(56.6)			46(26.3)	129(73.7)		
High level	44(43.6)	57(56.4)			24(22.9)	81(77.1)		
Education level								
Illiterate	3(75)	1(25)	**0.004**	**0.001**	1(25)	3(75)	0.11	0.20
Under diploma	26(55.3)	21(44.7)			16(33.3)	32(66.7)		
Diploma	78(56.1)	61(43.9)			45(30.8)	101(69.2)		
Master and higher	65(38)	106(62)			37(20.6)	143(79.4)		
Marital status								
Single	124(48.1)	134)51.9 (	0.80	0.91	28(26.2)	79(73.8)	0.99	0.86
Married	48)46.6 (	55)53.4 (			71(26.2)	200(73.8)		
Losing weight history								
yes	66(44)	84(56)	**0.01**	**0.01**	97(51.9)	90(48.1)	0.64	0.34
no	76(57.1)	57(42.9)			79(49.4)	81(50.6)		
rs 3,807,992 genotype								
AA+AG	89(44.9)	109(55.1)	0.10	0.15				
GG	42(56.0)	33(44.0)						

*EDII* energy-adjusted dietary inflammatory index, *BMI* body mass index, *WC* waist circumference, *HC* hip circumference, *NC* Neck circumference, *WHR* waist-hip ratio, *WHtR* waist-hight ratio, *BFM* body fat mass, *FFM* fat-free mass, *SMM* skeletal muscle mass, *SLM* soft lean mass, *BMC* Bone mineral content, *ECW* Extracellular water, *ICW* intracellular water, *FFMI* fat-free mass index, *FMI* fat mass index, *FBS* fasting blood glucose, *TC* total cholesterol, *HDL* high-density lipoprotein, *LDL* low-density lipoprotein

*P*-value: obtain from ANOVA

*P*-value ^*^: obtain from ANCOVA; adjusted for age, physical activity, total energy intake, and BMI

Continuous variables showed a MEAN ± standard error (SE), categorical variables showed as the number and (%)

*P* < 0.05 consider as significant, *P* = 0.06, and 0.07 consider as marginally significant

### Association between population characteristics across rs 3,807,992 genotypes and median EDII score

Associations between population characteristics across rs 3807992 genotypes and median EDII score are shown in Table 1. Age of starting obesity and history of losing weight was higher in upper vs. median group of EDII, and after controlling potential confounders, including age, BMI, energy intake, and physical activity, there was a marginally significant mean difference among the median of EDII (*P*=0.05). In the crude model, significant mean differences were found for physical activity, starting obesity age (*P*<0.05), whilst for body composition and biochemical variables in terms of fat-free mass (FFM), skeletal muscle mass (SMM), soft lean mass (SLM), fat-free mass index (FFMI), and HDL, there were also significant mean differences (*P*<0.05). Categorical variables, such as economic and education status, were significantly different across the median of EDII (*P*<0.05), moreover all the mentioned factors had a higher mean in the lower median of EDII. Also, EDII was associated with body fat mass (BFM) (*P*=0.05) and BFM (%) (*P*=0.01), bone mineral content (BMC) (*P*=0.04), trunk fat (*P*=0.01), visceral fat (*P*=0.03), fat mass index (FMI) (*P*=0.01), and marginally significantly different for TG (*P*=0.06).

### Association between population characteristics among genotype category

Subjects were divided into two groups according to risk alleles of CAV1 genotypes: GG (*n*=75) without risk alleles and AA+AG with risk alleles (*n*=198)_._ A marginal significant mean difference was found in visceral fat (*P*=0.05) and FMI (*P*=0.07) and HC (*P*=0.06) among CAV1 genotypes category in the crude model. There was a significant difference was found for TG (*P*=0.04) after adjustment among genotype category groups. Also, a significant mean difference for HDL remained stable across models (*P*=0.04) (Table 1).

### Galectin-3, leptin, and leptin resistance across rs 3,807,992 genotypes and median EDII score

**Fig. 1:**
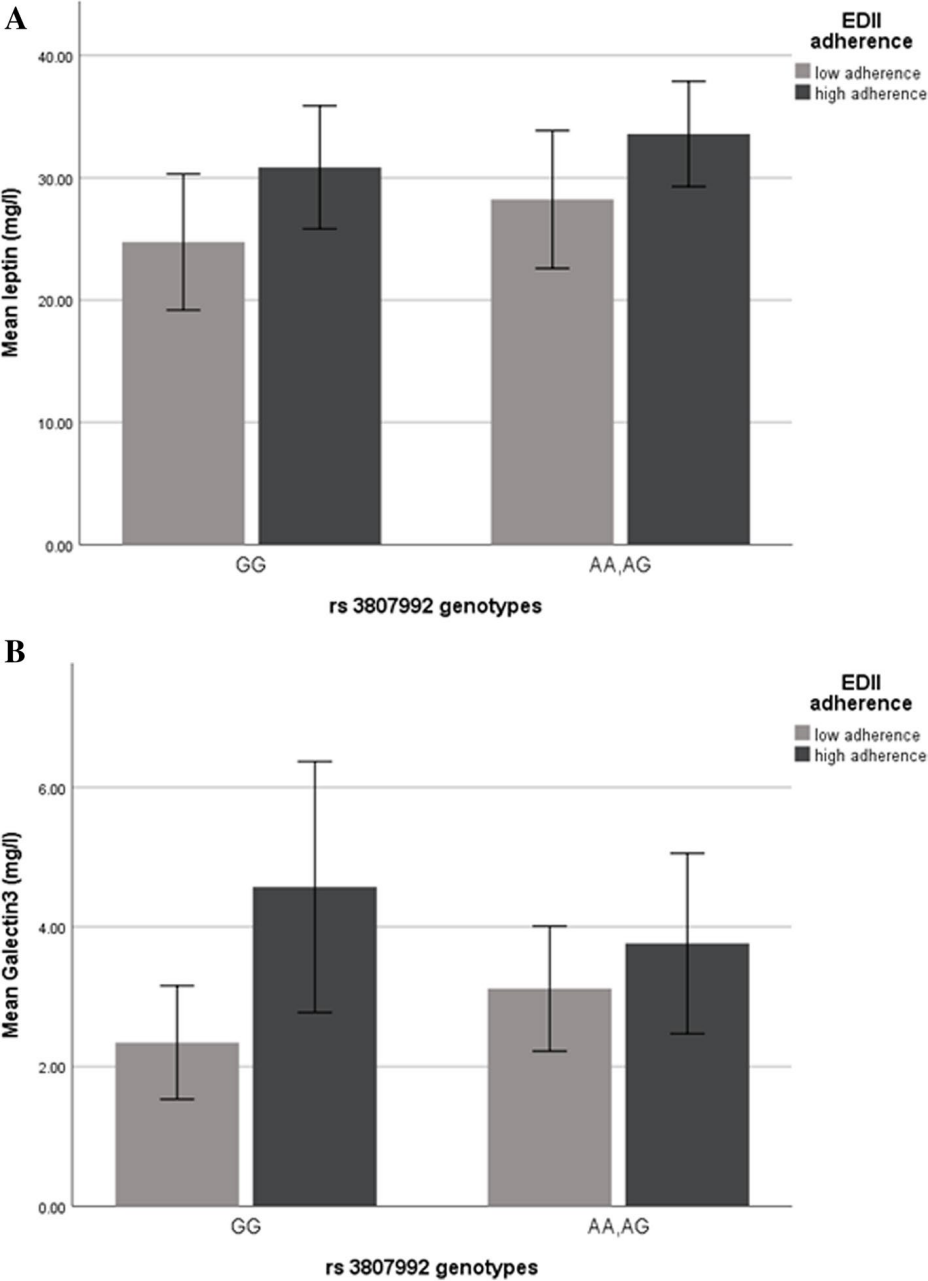
Food groups intakes among EDII categories in obese and overweight women

Although there was a significant mean difference among the EDII median for leptin (*P*=0.03), after further controlling with economic status and education, starting obesity age, there was no significant mean difference for leptin resistance (*P*=0.21). Moreover, a marginal significant mean difference was found for Galectin 3 (*P*=0.06) (Table 2). There was no significant mean difference among median of EDII in other variables (*P*>0.05). Food group intake of study population among EDII category are shown in Fig. 1.

**Table 2:** Galectin-3, leptin, and leptin resistance across rs 3,807,992 genotypes and median EDII score in obese and overweight women (*n* = 363)

Variables	EDII median		***P***-value	***p***-value*	rs 3,807,992 genotypes		***p***-value	***p***-value*
	<0.07	> = 0.07			GG	AA + AG		
	***N*** = 172	***N*** = 191			***N*** = 75	***N*** = 198		
	Mean ± SE				Mean ± SE			
Leptin (mg/l)	27.13 ± 3.45	29.87 ± 3.01	0.64	**0.03**	30.38 ± 2.82	26.69 ± 3.69	0.52	0.40
Leptin resistance	0.81 ± 0.05	0.91 ± 0.05	0.17	0.21	0.81 ± 0.06	0.90 ± 0.05	0.72	0.31
Galectin-3 (mg/l)	4.01 ± 1.88	4.99 ± 2.05	0.67	**0.06**	4.86 ± 1.43	2.73 ± 1.34	0.12	0.29

*EDII* energy-adjusted dietary inflammatory index, *SE* standard error

*P*-value: obtain from ANOVA

*P*-value ^*^: obtain from ANCOVA; adjusted for age, physical activity, total energy intake, BMI, economic status and education, age of starting obesity

*P* < 0.05 consider as significant, *P* = 0.06, and 0.07 consider as marginally significant

### The association between EDII score and with the leptin, leptin resistance and Galectin3

The association between EDII score and rs 3807992 genotypes with the leptin, leptin resistance, and Galactin3 are presented in Table 3. Increased adherence to EDII yielded an increase of 16.73 mg/l in leptin level (β= 16.73, 95% CI= 1.56, 39.3, *P*= 0.04) and 0.55 in leptin resistance (β=0.55, 95% CI=0.00, 1.30, *P*= 0.06) in model 2, which was adjusted for, economic status, education level, age of starting obesity, weight loss history. By contrast, increased adherence to EDII in the association with that genotype showed no significant association in Galectin3 in model 2 (β= 0.91, 95% CI= -0.64, 2.48, *P*=0.24) (Table 3).

**Table 3:** The association between EDII across and rs 3,807,992 genotypes on the leptin, and Galectin3 in obese and overweight (*n* = 363)

Variables	Models	EDII score		***P***-value^^*^	rs 3,807,992 genotypes		***P***-value^^*^
		β	95% CI		β	95% CI	
Leptin (mg/l)	**Crude**	2.34	−22.52,27.21	0.85^^^	1.32	−3.73,6.38	0.60
	**Model1**	14.03	−8.57,36.65	0.22^*^	−0.71	−5.4,3.91	0.76
	**Model2**	16.73	1.56,39.32	0.04^*^	2.53	−3.42,8.48	0.39
Leptin resistance	**Crude**	0.57	−0.36,1.51	0.22^^^	0.02	−1.24,0.17	0.72
	**Model1**	0.49	−0.24,1.23	0.18^*^	−0.01	−0.16,0.13	0.85
	**Model2**	0.55	0.00,1.30	0.06^*^	0.08	0.00,0.28	0.08
Galectin3 (mg/l)	**Crude**	21.45	−19.47,62.37	0.30^^^	−0.80	−3.93,2.31	0.60
	**Model1**	0.82	−0.59,2.25	0.25^*^	0.06	−3.84,3.97	0.97
	**Model2**	0.91	−0.64,2.48	0.24^*^	1.08	−3.40,5.58	0.62

*EDII* energy-adjusted dietary inflammatory index

Model 1: additionally adjusted for age, BMI, total energy intake, and physical activity

Model 2: additionally adjusted for, economic status, education level, age of starting obesity, losing weight history

^^^Significant level in the crude model

^*^Significant level after adjustment by Model1, 2

*P* < 0.05 consider as significant, *P* = 0.06, and 0.07 consider as marginally significant

### The association between rs 3,807,992 genotypes with the leptin, leptin resistance and Galectin3

After adjustment, there were not any significant association between CAV1 genotypes with a risk allele in the association with leptin (β=2.53, 95%CI= -3.42,8.48, *P*= 0.39), leptin resistance (β=0.08, 95%CI= 0.00,0.28, *P*=0.08), and Galectin 3 (β=1.08, 95%CI= -3.40,5.58, *P*=0.62) (Table 3).

### The interactions between adherence of EDII across rs 3,807,992 genotypes on the leptin, leptin resistance, and Galectin3

**Fig. 2:**
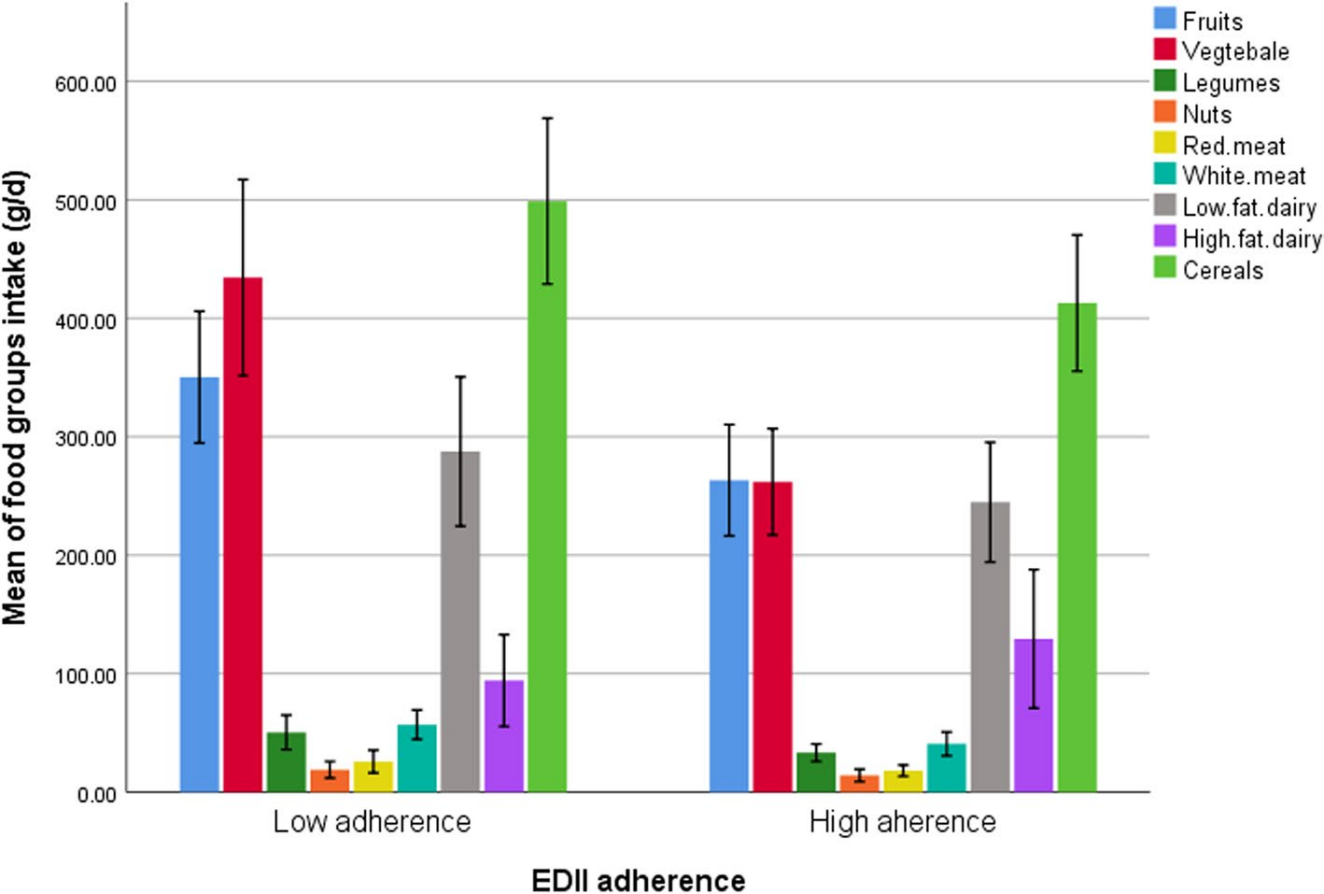
Interaction between rs 3,807,992 genotypes (GG consider as the reference group) with EDII on leptin and Galectin3 (**A**, **B** respectively). Vertical lines in every column are error bar, that have shown standard error. **A** Leptin (The *P*-value for AG + AA genotype: 0.02; *P*-value for adherence of EDII: 0.05; *P*-value for interaction between AG + AA genotype and Leptin: 0.65). **B** Galectin3 (The *P*-value for AG + AA genotype: 0.31; *P*-value for adherence of EDII: 0.02; *P*-value for interaction between AG + AA genotype and Galectin3:0.24)

The interactions between adherence of EDII across rs 3807992 genotypes on leptin, leptin resistance, and Galectin 3 were presented in Table 4. A marginal positive interaction was observed between EDII and risk alleles group (AA+AG) genotype in model 2 further adjustment by removing, economic status, education level more as confounders on leptin level (β=18.84, CI=3.25, 65.33, *P*=0.06) (Fig. 2). Increased adherence to EDII in the interaction with CAV1 genotypes containing risk alleles (AA+AG) leads to an increase in leptin level 79.15 mg/l (β= 79.15, CI= -1.23,163.94, *P*= 0.04) (Fig. 2) in model 3 after controlling for further potential confounders (age, BMI, total energy intake, and physical activity, economic status, education level, age of starting obesity, history of losing weight). By contrast, adherence to EDII in the interaction with the genotype that includes risk alleles (AA+AG) showed no significant interaction on leptin resistance not in the crude model (β=0.57, CI=-0.36,1.51, *P*=0.22), and even after adjustment in model 3 (β=0.55, CI= -0.99,2.09, *P*=0.48). Although no significant interaction was found between EDII and CAV1 genotypes on Galectin 3 in the crude model, there was a marginal positive interaction after adjustment in model 3 (β=31.35, CI=0.13,77.13, *P*= 0.05) (Fig. 2, Table 3).

**Table 4:** The interactions between adherence of EDII across rs 3,807,992 genotypes on the leptin, leptin resistance, and Galectin3 score in obese and overweight women (*n* = 363)

Variables	Models	EDII adherence^*^ AA + AG		
		Β	95% CI	***P***-value
Leptin (mg/l)	**Crude**	14.73	−41.06,70.54	0.60
	**Model1**	30.64	15.03,75.13	0.05
	**Model2**	18.84	3.25,65.33	**0.06**
	**Model3**	79.18	1.23,163.94	**0.04**
Leptin resistance	**Crude**	0.57	−0.36,1.51	**0.22**
	**Model1**	0.03	−1.94,2.01	**0.97**
	**Model2**	0.92	2.40,1.51	**0.45**
	**Model3**	0.55	−0.99,2.09	**0.48**
Galectin3 (mg/l)	**Crude**	21.45	−19.47,62.37	0.30
	**Model1**	24.16	−25.04,73.38	0.33
	**Model2**	0.15	0.01,1.03	0.07
	**Model3**	31.35	0.13,77.13	**0.05**

*EDII* energy-adjusted dietary inflammatory index

Model 1: additionally adjusted for age, BMI, total energy intake, and physical activity

Model 2: additionally adjusted for, economic status, education level

Model 3: further adjustment with the age of starting obesity, history of losing weight

GG genotype has 0 risk allele, AG genotype has one and AA genotype have two risk allele

GG genotype is considered as a reference group

^*^Significant level in the crude model and after adjustment by Model1, 2, and 3

*P* < 0.05 consider as significant, *P* = 0.06, and 0.07 consider as marginally significant

## Discussion

To the best of our knowledge, the present study is the first cross-sectional study to investigate the interaction of EDII and CAV1 genotypes on leptin, leptin resistance, and Galectin 3 as outcomes. We found that, after taking into account confounding variables, increased adherence to EDII, in association with leptin level and leptin resistance, is associated wi5h an increase in both variables. Also, our results suggest that EDII interacts positively with CAV1 genotypes including risk alleles (AA+AG) on leptin level and Galectin 3.

Leptin is considered a cytokine that is created by adipocytes, and it leads to the induction of inflammation. The pro-inflammatory properties of leptin have been proposed to be comprable to those of immune cell-derived cytokines, including interleukin 6 (IL-6) and tumor necrosis factor-alpha (TNF-α) [31, 32]. Present study has indicated EDII was associated with leptin, and leptin resistance. Similar to our study Muhammad et al. showed that the DII score was positively associated with plasma leptin concentration [33], which was not in line with a cross-sectional study in the USA that demonstrated the DII score was not related to leptin [34]. According to an observational study among children, exposure to a more pro-inflammatory diet in boys and girls is associated with an increase in leptin concentrations [35]. It has been illustrated that leptin resistance may depend on the variety of nutrients consumed from the diet, supporting the hypothesis of the vital role of an accurate distribution in the dietary pattern. Recent studies have contributed to recognizing the mechanisms regarding the impaired response to leptin, and desensitization of leptin receptor, down-regulation of its intracellular signaling and inflammation are regarded as key routes involved [36]. The DII not only includes micronutrients and macronutrients, but also includes general intake of bioactive components containing flavonoids, spices, and tea. The whole score takes into account the complete diet, not just separate nutrients or foods [21]. Thus, inflammatory factors are affected by bioactive components [37]. Unclear processes appear underlie how a pro-inflammatory diet causes obesity. Previous research has shown that early in the process leading to weight gain, substantial amounts of inflammatory biomarkers are present. In Duncan et al., it was discovered that during a 3-year period in middle-aged people, fibrinogen, leukocytes, and other indicators of chronic low-grade inflammation engendered weight gain [38, 39]. Increased levels of inflammation-sensitive plasma proteins in middle-aged men from the cohort of the Malmo Preventive Study indicated weight increase, even in middle-aged and older participants' inflammatory indicators, were directly linked to weight increase [40–42]. Moreover, in Ramallal. et al, the authors indicated the risk of acquiring new-onset overweight or obesity and yearly weight gain were both significantly increased by proinflammatory diet [43]. Uncertain processes underlie how a proinflammatory diet promotes obesity, however, there is some evidential mechanism in the tendency to acquire weight. Proinflammatory cytokines including IL-6, IL-1, and TNF-a may increase appetite, resulting in an increase in caloric intake and fat storage, B-adrenergic receptors brought on by persistent peripheral sympathetic nervous system stimulation brought on by adiposity signals like leptin and insulin, which are linked to the inflammatory process [44–46]. Moreover, excess consumption of some nutrients may also produce hypothalamus inflammation, and impact of nutrition on alterations in the gut microbiota, which come before the low-grade inflammation that encourages adiposity, represent another putative mechanism [47–49]. Blood leptin levels and body fat are shown to be associated, where more adiposity equates to higher leptin levels. This appears to represent the start of the defect loop of inflammation by diet in the increment of leptin level [50]. In the present study, due to the non-significance of the mean difference weight among median of EDII, but the percentage BFM weight was higher in subjects with more adherence of EDII, which can highlight the role of this mechanism. Leptin resistance is a broad-based pathophysiological condition. The leptin axis interacts in a useful way with factors involved in metabolism, such as insulin, and inflammation, including innate immune system mediators, such as interleukin-6. Leptin resistance is reported to be caused by a physical interaction between leptin and C-reactive protein [51]. Therefore, it seems that following an inflammatory diet with its effect on the state of inflammatory factors and insulin resistance can be effective in creating leptin resistance [52].The present study demonstrates that increased adherence to EDII in the association leptin and leptin resistance leads to an increase in leptin level and leptin resistance; however, there was no impact on Galectin 3. Galectin 3 is a crucial component of several physiological processes, including cell adhesion, proliferation, apoptosis, signal transduction, and the control of immunological and inflammatory responses [53, 54]. Numerous types of human cells, including immunological and inflammatory cells, fibroblasts, endothelium, and epithelium, are highly expressed in this protein [55]. Galectin-3’s function in determining nutritional status has not previously been investigated.

In the present study, there was no association between CAV1 and leptin, leptin resistance, and Galectin3. CAV1 is considered a key factor with cellular functions including pinocytosis and regulation of cell signaling [56]. Singh et al. present a feedback loop of CAV1 mediated downregulation of leptin signaling [16]. The significant and novel findings pointed out that there is a strong association between leptin and CAV1, so it contributes to raising CAV1 expression which impairs the signaling of leptin. Also, the associated mechanisms need to be ascertained [57]. In line with our findings, Schroeter et al. found that Caveolin deficiency was associated with the lack of leptin in vivo [58]. In this study, we found no association between caveolin with leptin, leptin resistance, and Galectin 3; by contrast, some studies have demonstrated that Galectin3 induces integrin function and Caveolin phosphorylation, suggesting that Galectin 3 may be related to Caveolin [59, 60].

We found that, after taking into account confounding variables, increased adherence to EDII in the interaction with CAV1 genotype including risk alleles (AA+AG) leads to a positive interaction on leptin level and Galectin 3. Inflammatory stimuli can lead to raised Caveolin expression by inhibiting upstream regulators of antioxidant defense enzymes; Caveolin expression can further intensify the inflammatory reaction. Caveolae nutritional modulation may apply an opportunity to upset inflammatory signaling events; indeed, DII has strong anti-inflammatory effects which occur through modifications to the lipid microenvironment [61].

The major strength of this investigation is that it is the first study to evaluate the interaction of EDII and CAV1 genotypes on leptin, leptin resistance, and Galectin3. These interactions remained significant across multiple testing, despite the high correlations among the outcomes of interest. Additionally, demographic characteristics and FFQ were measured/collected by a trained dietitian. Moreover, the knowledge gained from this study may be applied to clinical practice and contribute to personalized therapies for the prevention and treatment of metabolic disorders.

Limitations of this investigation include the use of cross-sectional design, which precludes causal inferences. Longitudinal epidemiologic studies and biochemical experimentally based research are required to elucidate and strengthen the findings from this study. Furthermore, whether this interaction relates to changes in CAV1 genotypes has not been explored. Besides, the pathway linking dietary inflammatory index to this CAV1 gene is unknown. Also, FFQ was used for recording the subject’s food, which is subject to memory and recall bias. Although the FFQ is typically used to examine long-term dietary consumptions, its closed structure with limited response options limits its ability to determine between-person variations, so there can be some misclassification. Residual confounding for is another limitation. Moreover, we did not measure cytokines in participants, and only measured leptin and Galectin-3. Because in the initial approach, we did not have a presupposition for this kind of influence, and our null hypothesis only pertained to the presence or absence of association and interaction between rs 3807992 genotypes with the dietary inflammatory index on leptin, leptin resistance, and Galectin 3, and not the mechanism of effect or the investigation of the cause of this influence. However, it seems that there is a need to conduct more studies in this field to investigate these inflammatory factors and the serum levels of these cytokines and It is their effectiveness.

## Conclusion

We found that there may be an association between DII and leptin and leptin resistance, and that high adherence of EDII and CAV1 genotypes containing risk alleles have prognostic value and are associated with increases in both leptin and Galectin3, but not leptin resistance Nevertheless, prospective investigation of the interaction between EDII and CAV1 genotypes should be a priority in further study of this interaction.

## Data Availability

The data that support the findings of this study are available from correspond author but restrictions apply to the availability of these data, which were used under license for the current study, and so are not publicly available. Data are however available from the authors upon reasonable request and with permission of correspond author.

## Abbreviations

NCDs: Non-communicable diseases
CAV1: Caveolin1
DII: Dietary Inflammatory Index
CVDs: Cardiovascular diseases
BMI: Body mass index
FFM: Fat-free mass
WC: Waist circumference
HC: Hip circumference
WHR: Waist-to-hip ratio
WHtR: Waist-to-height ratio
NC: Neck diameter
EDII: Energy-adjusted DII
FFQ: Food frequency questionnaire
TG: Triglyceride
TC: Total cholesterol
HDL: High-density lipoprotein
LDL: Low-density lipoprotein
FBS: Fasting blood pressure
PCR-RFLP: polymerase chain reaction-restriction fragment length polymorphism
ELISA: Enzyme-linked immunosorbent assay
HOMA: Homeostatic model assessment
SBP: Systolic blood pressure
DBP: Diastolic blood pressure
IPAQ: International Physical Activity Questionnaire
SD: Standard deviation
ANCOVA: Analysis of covariance
SMM: Skeletal muscle mass
SLM: Soft lean mass
ECW: Extracellular water
ICW: Intracellular water
FFMI: Fat-free mass index
IL-6: Interleukin 6
TNF-α: Tumor necrosis factor-alph

## References

[1] James WPT (2008). The epidemiology of obesity: the size of the problem. J Intern Med.

[2] Djalalinia S (2020). Patterns of obesity and overweight in the Iranian population: findings of STEPs 2016. Front Endocrinol.

[3] Renner B (2012). Why we eat what we eat. The eating motivation survey (TEMS). Appetite.

[4] Pankow JS (2001). Familial and genetic determinants of systemic markers of inflammation: the NHLBI family heart study. Atherosclerosis.

[5] Flegal KM (2002). Prevalence and trends in obesity among US adults, 1999-2000. JAMA.

[6] Chooi YC, Ding C, Magkos F (2019). The epidemiology of obesity. Metabolism.

[7] Monteiro R, Azevedo I (2010). Chronic inflammation in obesity and the metabolic syndrome. Mediat Inflamm.

[8] Han JM, Levings MK (2013). Immune regulation in obesity-associated adipose inflammation. J Immunol.

[9] Morris DL, Singer K, Lumeng CN (2011). Adipose tissue macrophages: phenotypic plasticity and diversity in lean and obese states. Curr Opin Clin Nutr Metab Care.

[10] Considine RV (1996). Serum immunoreactive-leptin concentrations in normal-weight and obese humans. N Engl J Med.

[11] Pelleymounter MA (1995). Effects of the obese gene product on body weight regulation in Ob/Ob mice. Science.

[12] Brierley AS (2002). Antarctic krill under sea ice: elevated abundance in a narrow band just south of ice edge. Science.

[13] Lam NT (2004). Leptin increases hepatic insulin sensitivity and protein tyrosine phosphatase 1B expression. Mol Endocrinol.

[14] Liu F-T, Hsu DK (2007). The role of galectin-3 in promotion of the inflammatory response. Drug News Perspect.

[15] Henderson NC, Sethi T (2009). The regulation of inflammation by galectin-3. Immunol Rev.

[16] Singh P (2011). Leptin upregulates caveolin-1 expression: implications for development of atherosclerosis. Atherosclerosis.

[17] Fielding CJ, Fielding PE (2001). Caveolae and intracellular trafficking of cholesterol. Adv Drug Deliv Rev.

[18] Shyu H-Y (2017). Association of eNOS and Cav-1 gene polymorphisms with susceptibility risk of large artery atherosclerotic stroke. PLoS One.

[19] Jasmin J-F (2006). SOCS proteins and caveolin-1 as negative regulators of endocrine signaling. Trends Endocrinol Metab.

[20] Cavicchia PP (2009). A new dietary inflammatory index predicts interval changes in serum high-sensitivity C-reactive protein. J Nutr.

[21] Shivappa N (2014). Designing and developing a literature-derived, population-based dietary inflammatory index. Public Health Nutr.

[22] Shivappa N (2014). A population-based dietary inflammatory index predicts levels of C-reactive protein in the seasonal variation of blood cholesterol study (SEASONS). Public Health Nutr.

[23] Azizi F (2002). Distribution of blood pressure and prevalence of hypertension in Tehran adult population: Tehran lipid and glucose study (TLGS), 1999–2000. J Hum Hypertens.

[24] Lee J, Reed D, Price R (2001). Leptin resistance is associated with extreme obesity and aggregates in families. Int J Obes.

[25] Graf S (2015). Evaluation of three indirect calorimetry devices in mechanically ventilated patients: which device compares best with the Deltatrac II®? A prospective observational study. Clin Nutr.

[26] Neelakantan N (2016). Development of a semi-quantitative food frequency questionnaire to assess the dietary intake of a multi-ethnic urban Asian population. Nutrients.

[27] Rezazadeh A, Omidvar N, Tucker KL (2020). Food frequency questionnaires developed and validated in Iran: a systematic review. Epidemiol Health.

[28] Ghaffarpour M, Houshiar-Rad A, Kianfar H (1999). The manual for household measures, cooking yields factors and edible portion of foods. Tehran: Nashre Olume Keshavarzy.

[29] Tanabe N (2009). Risk assessment by post-challenge plasma glucose, insulin response ratio, and other indices of insulin resistance and/or secretion for predicting the development of type 2 diabetes. Intern Med.

[30] Craig CL (2003). International physical activity questionnaire: 12-country reliability and validity. Med Sci Sports Exerc.

[31] Iikuni N (2008). Leptin and inflammation. Curr Immunol Rev.

[32] Wellen KE, Hotamisligil GS (2005). Inflammation, stress, and diabetes. J Clin Invest.

[33] Muhammad HFL (2019). Dietary inflammatory index score and its association with body weight, blood pressure, lipid profile, and leptin in Indonesian adults. Nutrients.

[34] Sokol A (2016). Association between the dietary inflammatory index, waist-to-hip ratio and metabolic syndrome. Nutr Res.

[35] Barragán-Vázquez S (2020). Pro-inflammatory diet is associated with adiposity during childhood and with Adipokines and inflammatory markers at 11 years in Mexican children. Nutrients.

[36] Sáinz N (2015). Leptin resistance and diet-induced obesity: central and peripheral actions of leptin. Metabolism.

[37] Shivappa N (2015). Associations between dietary inflammatory index and inflammatory markers in the Asklepios study. Br J Nutr.

[38] Duncan BB (2000). Fibrinogen, other putative markers of inflammation, and weight gain in middle-aged adults—the ARIC study. Obes Res.

[39] Duncan BB (2003). Inflammation markers predict increased weight gain in smoking quitters. Obes Res.

[40] Engstrom G (2003). Inflammation-sensitive plasma proteins are associated with future weight gain. Diabetes.

[41] Barzilay J (2006). The association of markers of inflammation with weight change in older adults: the cardiovascular health study. Int J Obes.

[42] Holz T (2010). Markers of inflammation and weight change in middle-aged adults: results from the prospective MONICA/KORA S3/F3 study. Obesity.

[43] Ramallal R (2017). Inflammatory potential of diet, weight gain, and incidence of overweight/obesity: the SUN cohort. Obesity.

[44] Inui A, Meguid MM (2003). Cachexia and obesity: two sides of one coin?. Curr Opin Clin Nutr Metab Care.

[45] Seals DR, Bell C (2004). Chronic sympathetic activation: consequence and cause of age-associated obesity?. Diabetes.

[46] Lohse M (1996). Mechanisms of β-adrenergic receptor desensitization: from molecular biology to heart failure. Basic Res Cardiol.

[47] Thaler JP (2013). Hypothalamic inflammation: marker or mechanism of obesity pathogenesis?. Diabetes.

[48] Chassaing B, Gewirtz AT (2016). Has provoking microbiota aggression driven the obesity epidemic?. Bioessays.

[49] Turnbaugh PJ (2006). An obesity-associated gut microbiome with increased capacity for energy harvest. nature.

[50] Benoit SC (2004). Insulin and leptin as adiposity signals. Recent Prog Horm Res.

[51] Martin SS, Qasim A, Reilly MP (2008). Leptin resistance: a possible interface of inflammation and metabolism in obesity-related cardiovascular disease. J Am Coll Cardiol.

[52] Carvalho CA (2019). The dietary inflammatory index and insulin resistance or metabolic syndrome in young adults. Nutrition.

[53] Thiemann S, Baum LG (2016). Galectins and immune responses—just how do they do those things they do?. Annu Rev Immunol.

[54] Liu F-T, Rabinovich GA (2005). Galectins as modulators of tumour progression. Nat Rev Cancer.

[55] Sciacchitano S (2018). Galectin-3: one molecule for an alphabet of diseases, from a to Z. Int J Mol Sci.

[56] Gratton J-P, Bernatchez P, Sessa WC (2004). Caveolae and caveolins in the cardiovascular system. Circ Res.

[57] Devaraj S, Torok N (2011). Leptin: the missing link between obesity and heart disease?. Atherosclerosis.

[58] Schroeter MR (2013). Leptin promotes neointima formation and smooth muscle cell proliferation via NADPH oxidase activation and signalling in caveolin-rich microdomains. Cardiovasc Res.

[59] Lagana A (2006). Galectin binding to Mgat5-modified N-glycans regulates fibronectin matrix remodeling in tumor cells. Mol Cell Biol.

[60] Goetz JG (2008). Concerted regulation of focal adhesion dynamics by galectin-3 and tyrosine-phosphorylated caveolin-1. J Cell Biol.

[61] Layne J (2011). Caveolae: a regulatory platform for nutritional modulation of inflammatory diseases. J Nutr Biochem.

